# *In silico* functional dissection of saturation mutagenesis: Interpreting the relationship between phenotypes and changes in protein stability, interactions and activity

**DOI:** 10.1038/srep19848

**Published:** 2016-01-22

**Authors:** Douglas E. V. Pires, Jing Chen, Tom L. Blundell, David B. Ascher

**Affiliations:** 1 Department of Biochemistry, Sanger Building, University of Cambridge, 80 Tennis Court Road, Cambridge, CB2 1GA, UK; 2 Centro de Pesquisas René Rachou, Fundação Oswaldo Cruz, Avenida Augusto de Lima 1715, Belo Horizonte, 30190-002, Brazil

## Abstract

Despite interest in associating polymorphisms with clinical or experimental phenotypes, functional interpretation of mutation data has lagged behind generation of data from modern high-throughput techniques and the accurate prediction of the molecular impact of a mutation remains a non-trivial task. We present here an integrated knowledge-driven computational workflow designed to evaluate the effects of experimental and disease missense mutations on protein structure and interactions. We exemplify its application with analyses of saturation mutagenesis of DBR1 and Gal4 and show that the experimental phenotypes for over 80% of the mutations correlate well with predicted effects of mutations on protein stability and RNA binding affinity. We also show that analysis of mutations in VHL using our workflow provides valuable insights into the effects of mutations, and their links to the risk of developing renal carcinoma. Taken together the analyses of the three examples demonstrate that structural bioinformatics tools, when applied in a systematic, integrated way, can rapidly analyse a given system to provide a powerful approach for predicting structural and functional effects of thousands of mutations in order to reveal molecular mechanisms leading to a phenotype. Missense or non-synonymous mutations are nucleotide substitutions that alter the amino acid sequence of a protein. Their effects can range from modifying transcription, translation, processing and splicing, localization, changing stability of the protein, altering its dynamics or interactions with other proteins, nucleic acids and ligands, including small molecules and metal ions. The advent of high-throughput techniques including sequencing and saturation mutagenesis has provided large amounts of phenotypic data linked to mutations. However, one of the hurdles has been understanding and quantifying the effects of a particular mutation, and how they translate into a given phenotype. One approach to overcome this is to use robust, accurate and scalable computational methods to understand and correlate structural effects of mutations with disease.

Over the past twenty years, multiple *in silico* approaches to predict how mutations affect protein stability have been developed based on various evolutionary and physicochemical hypotheses. These include methods that seek to understand the effects of amino acid substitutions from the protein sequence alone, and those that exploit the extensive structural information now available for many proteins. The sequence-based approaches include, amongst others, the well established and widely used methods SIFT^1^ and PolyPhen^2^. Our lab developed one of the pioneering structure-based approaches, SDM^3^,^4^, which uses environment-specific substitution tables of protein families to derive a statistical potential energy function. Subsequently, *in silico* methods based on a variety of evolutionary and physical chemical hypotheses have been proposed for predicting the effects of mutations on protein stability^3^,^5^,^6^,^7^,^8^,^9^,^10^. More recently, we have used machine learning and graph-based signatures to represent the three-dimensional environment of the wild-type residue and have developed mCSM-Stability, which quantitatively predicts the change upon mutation in the Gibbs free energy (ΔΔG) of folding^11^. By combining these two different approaches we were able to develop an optimized consensus method, DUET, which takes advantage of their relative strengths^12^.

To date, significantly less attention has been focused on understanding the effects of mutations on the recognition of binding partners, including proteins, nucleic acids and other ligands. These properties are much more difficult to predict from the amino acid sequence alone. Several methods have been recently proposed in an attempt to understand how mutations on protein interfaces affect binding affinity^9^,^13^,^14^,^15^,^16^, although there is still significant room for improvement^17^. In order to bridge this gap, we have developed methods based on graph signatures to predict accurately changes upon mutation in protein-protein (mCSM-PPI) and protein-nucleic acid (mCSM-NA) affinities^11^, and more recently efforts to predict the changes in protein-ligand affinity (mCSM-Lig)^18^.

Recent advances in experimental approaches have integrated saturation mutagenesis coupled to a biological assay output as a tool to facilitate the high-resolution, functional dissection of mutations^19^,^20^. However, understanding the functional consequence of these mutations, and how they are linked to the experimental phenotype, remains a very complex and challenging task. The effects of mutations can be complex and multifactorial. Here we present a knowledge-driven computational workflow that can be easily implemented in a pipeline to analyze the structural and functional effects of mutations (Fig. 1). This approach is contingent upon a good understanding of the protein (both structure and function) and the system being mutated, as highlighted at the top of Fig. 1. The workflow then uses structural methods to explore the molecular mechanisms of mutations and their links to the biological effects experimentally observed.

## The lariat debranching enzyme DBR1

Findlay and colleagues reported in Nature a CRISPR/Cas9 cleavage system coupled with multiplex homology-directed repair to perform saturation editing of a conserved 25 amino acid region of the RNA lariat debranching enzyme DBR1, an essential gene. They coupled this to a growth-based assay to evaluate the phenotypic effects of over 170 distinct missense mutations. The authors noted a weak correlation between the sequence-based predictor CADD^21^ and PolyPhen-2^22^ with the phenotypic output. This region is quite highly conserved, leading to PolyPhen and CADD predicting most mutations as damaging^19^. We therefore applied our workflow in an attempt to analyse the molecular mechanism underlying the phenotypic effects of these mutations (Figure S1).

Figure S2 shows the growth response distribution (average log_2_-enrichment score between replicates) of the 172 missense mutations reported by Findlay and colleagues^19^ and considered in this analysis, ranging from residues 84 to 108 of human DBR1. There is a bimodal distribution of the phenotypic effects of the mutations, with a distinct division of the experimental data into two different (and balanced) groups: a) minimum or no reduced growth (on the right) and b) highly reduced growth (on the left). Mutations were classified in one of the two groups accordingly, using an average log-enrichment score threshold of −3. Due to the essential nature of DBR1, and the design of the experiments, the change of growth could be directly related to the point mutations in DBR1^19^.

**Figure 2:**
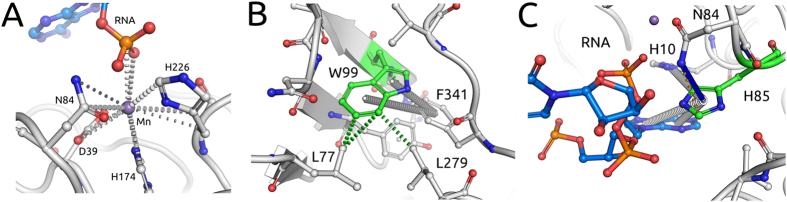
Noncovalent interaction networks in DBR1. (**A**) shows depicts interactions between Manganese ion and the DRB1-RNA complex. The ion is coordinated by a series of interactions within the protein as well as with the RNA molecule. Mutations on these residues would, therefore, disrupt manganese binding, affecting catalytic activity directly. (**B,C**) depicts noncovalent interaction network of mutated residues in the DRB1-RNA complex. Mutated residues are depicted in green and the RNA fragment in blue. Hydrogen bonds are depicted as red dotted lines, hydrophobic interactions in green and ring-ring interactions in grey. Panel B shows residue Trp99 performing a series of hydrophobic and ring interactions. Mutation from tryptophan to glycine would destabilize the protein given the loss of interactions leading to a loss of entropy when folding. Panel C shows residue His85, whose mutations are predicted to also affect RNA binding affinity. His85 makes a series of inter and intramolecular ring interactions that would be lost by a mutation to serine.

The workflow described here to study the effects of mutations relies upon the availability of high quality experimental or predicted protein structures. The DBR1 family is highly conserved and redundant, with human DBR1 previously shown to rescue yeast mutants^23^. As no experimental structure of DBR1 was available, high-confidence homology models were built of residues 2-349 of the apo- and RNA-bound protein. This region of DBR1 adopts an MPE fold^24^. The crystal structures of the homologue DCR1 (PDB codes: 4PEF, 4PEH and 4PEI^24^) were used to identify the location of the catalytic manganese (Fig. 2A) and the RNA binding site.

### Manganese Coordinating Residues

DBR1 is a phosphodiesterase with a catalytic manganese coordinated by Asp9, Asn84, His174 and His226 (Fig. 2A). It would be expected that mutations affecting one of these residues would disrupt manganese binding, and hence activity, leading to reduced growth. Among the mutations analyzed, 19 (11%) were of Asn84. As predicted these were all associated with very low log-enrichment scores and hence greatly reduced growth (p <0.001 by two-tailed Z-test). Analysis by mCSM-metal confirmed all mutations of Asn84, but no others, were deleterious to manganese binding. As these mutations could be explained from the expected altered catalytic activity, they were therefore removed from the structural analyses below.

### Predicted Highly Destabilizing Mutations

Using the apo model of DBR1, as well as that bound to an RNA substrate analogue, the change in stability caused by the missense mutations was quantitatively predicted by mCSM-Stability, SDM and DUET. Amongst the mutations, 27 (17% of the non-catalytic mutations) were predicted to be highly destabilizing (ΔΔG < −2 Kcal/mol) by mCSM-Stability, for example see Fig. 2B. These were associated with reduced growth (p = 0.037 by two-tailed Z-test), with 70% having average log-enrichment scores lower than −3.

### Mutations Predicted to Reduce PPI Affinity

Since the protein interacting partners of DBR1 have not yet been characterized, mCSM-PPI^11^ could not be used to assess the effects of the mutations upon their interactions. In addition, the residues mutated were either buried or located in the RNA-binding region. Furthermore, analysis with Crescendo did not support a role of these residues in mediating a protein-protein interaction^25^.

### Mutations Predicted to Greatly Reduce RNA Affinity

We used the model of DBR1 in complex with an RNA substrate analogue and mCSM-NA to predict the effect of the mutations upon RNA binding affinity. mCSM-NA predicted 25 mutations (16% of the non-catalytic mutations) to be highly destabilizing to RNA binding (ΔΔG < −2 Kcal/mol). These mutations presented a clear reduction in cell growth (p = 0.011 by the two-tailed Z-test), with 76% having a log-enrichment score below −3. This effect is exemplified by the interactions that some of the wild-type residues make in the RNA binding pocket (Fig. 2C). There was some overlap with the previous group of mutations, as 13 of these mutations (44%) were also predicted to be highly destabilizing by mCSM-Stability. By integrating these two predicted effects we were able to train a binary classifier based on Random Forest that classified correctly the phenotype of all but one mutation, suggesting that a combination of the two predictive outputs can be used to explain phenotypic results.

### Mutations Predicted to Have Mild Effects on Stability or RNA Binding

We were, however, interested in exploring further those 112 missense mutations that were not predicted to have a large effect either on stability or RNA binding. Neither changes in protein stability nor RNA affinity, taken separately, correlated strongly enough with the phenotypic results to explain their mechanism (ρ = 0.22 for mCSM-Stability and ρ = 0.48 for mCSM-NA).

**Figure 3:**
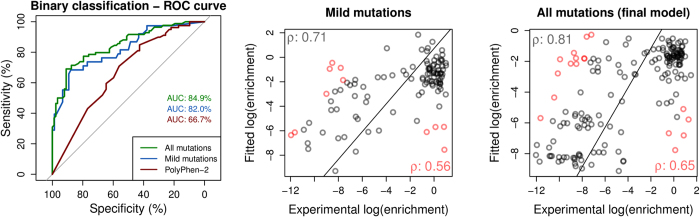
Performance analysis on classification and regression models of the phenotypic effects of DBR1 mutations. The left-hand graph shows the ROC curves for the binary classifiers trained with stability (DUET, SDM and mCSM-Stability) and RNA binding affinity change (mCSM-NA) predictions. Three curves are shown with the performance for the developed classifier on the complete set of mutations, the set of mild mutations and the performance of PolyPhen-2 on a selected set of mutations. The area under the curve values (AUC) for each classifier are also shown. The remaining graphs show regression results for those mutations not predicted to have large effects on stability or RNA binding (center graph) and for the final model including all mutations (right graph). Fitted log(enrichment) scores using DUET, SDM, mCSM-Stability and mCSM-NA are combined using linear equations compared to the average phenotypic results obtained by Gregory *et al.* (2014). For each data set the Pearson’s correlation coefficient (ρ) is shown in the bottom-right part of each graph and at the top-left after 10% outlier removal. Outliers are depicted in red.

In theory mutations could destabilize the protein, alter RNA binding or a combination of the two. Therefore the predicted changes in stability and RNA binding affinity from mCSM were used to train a binary classification model to predict whether a given mutation resulted in reduced growth. This achieved an AUC = 0.82, with accuracy of 78% (combined using the Random Forest algorithm^26^, evaluated by leave-one-out cross validation), as shown in the ROC curve on Fig. 3, left graph. Also by combining the predictions from DUET, mCSM-Stability and mCSM-NA, using a simple linear Regression Model Tree^27^, we observed a correlation with the average phenotypic results (ρ = 0.56. Fig. 3, center graph).

This suggests that the phenotypic results of approximately 80% of the DBR1 mutations that cannot be explained by large changes in stability, RNA affinity or metal binding, can be explained by changes in RNA binding affinity and protein stability together. This is consistent with the idea that missense mutations can have a multitude of effects on a protein function.

### Categorical Analysis of All DBR1 Experimental Mutations

No clear correlation between the experimental measurement and stability changes alone was initially observed on the full data set (ρ < 0.1 for mCSM-Stability and DUET and ρ = 0.14 for SDM). A weak correlation of ρ = 0.35 was observed between the predicted effects on RNA affinity by mCSM-NA and the experimentally measured reduced growth, higher than observed by the authors using CADD (ρ = 0.30).

Ideally we wished to generate a model that could explain the phenotypic effects of most of the mutations characterized by Findlay and colleagues^19^. We had observed with the mild mutations that taking into consideration effects on both stability and RNA affinity could explain a majority of phenotypic effects. Therefore, using mCSM-NA and stability predictions and by flagging the metal coordinating residues, we trained a binary classifier using the Random Forest algorithm^26^. We were able to achieve an accuracy of 78% with an AUC = 0.85 (evaluated by leave-one-out cross validation), significantly higher than the performance achieved by the authors using PolyPhen-2 (AUC = 0.667, over a reduced set of 160 missense mutations), as depicted on Fig. 3, left graph. This suggested that the phenotypic effects of over three quarters of the mutations could be explained by alterations in protein stability and/or RNA binding affinity and predicted using mCSM-Stability, SDM, DUET and mCSM-NA.

### Regression Analysis of All DBR1 Experimental Mutations

**Figure 4:**
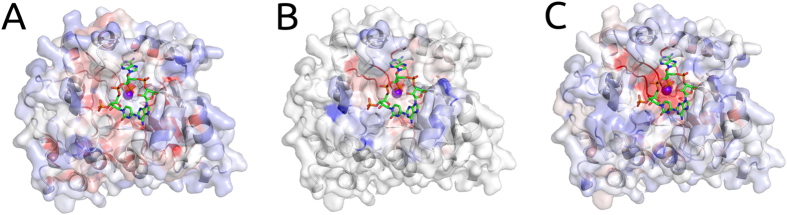
Heatmap of the average predicted changes upon mutation on DBR1. The figure shows the average prediction per residue (considering all 19 potential mutations at each position) in stability (left), RNA affinity (middle) and enrichment score (right). Residues were coloured in a scale from blue to red indicating the average effect from stabilizing to destabilizing as predicted by mCSM-Stability, mCSM-NA or the degree of reduced cell growth as predicted by the final regression model.

Using a Regression Model Tree^27^, we combined the predictions from SDM, mCSM-Stability and mCSM-NA together with the flag for catalytic residues in a consensus score, generating linear models that can describe different partitions of the data, better associating patterns between the *in silico* analysis with the phenotypic outcome. A correlation of ρ = 0.64 on 10-fold cross validation and ρ = 0.65 on leave-one-out cross validation was obtained (Fig. 3, right graph). This could be extrapolated to predict the expected phenotypic effects of mutations to the rest of the model- allowing rapid identification of regions of potential interest (Fig. 4). This highlights, as expected, that key residues involved in manganese coordination and RNA binding are likely to have the greatest effects on cell growth.

Interestingly, the outlier mutations tended to have very low log-enrichment scores (Fig. S3). While it is typical for extreme effects to be poorly fitted by machine-learning methods, it is also possible that the observed reduced growth of these mutations could be due to other effects that we have not considered here, for example changes in levels of transcription or translation. A complete prediction performance comparison before and after 10% outlier removal is available on Table S1 of Supplementary Material.

## The yeast transcription factor Gal4

Kitzman and colleagues^20^ presented in Nature Methods a programmed allelic series saturation mutagenesis of residues 2–64 of the yeast transcription factor Gal4, essential for the metabolism of galactose and melibiose. This was coupled to a reporter assay, which allowed them to link the effects of 1083 missense mutations to changes in cell growth in the presence of a selectable marker. A histogram of the effects of the mutations in growth response distribution (log_2_-enrichment) is shown in Figure S4, with those mutations presenting a log-enrichment score of lower than −3 classified as leading to reduced growth. In an attempt to analyse the molecular mechanism underlying the phenotypic effects of these mutations we applied our workflow (Figure S5). The X-ray crystal structure of Gal4 from Saccharomyces cerevisiae in complex with DNA has been solved (PDB ID: 3COQ^28^), and was used for this analysis.

### Zinc coordinating residues

**Figure 5:**
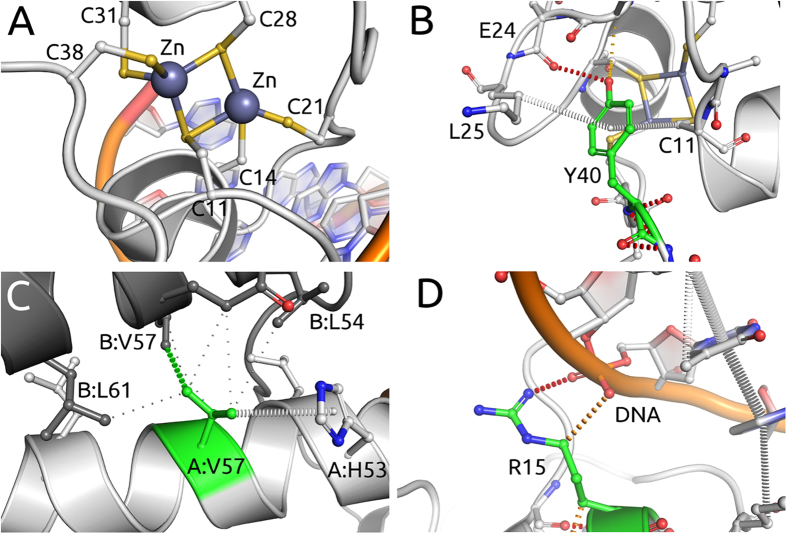
Noncovalent interaction networks in Gal4. (**A**) depicts interactions between a pair of Zn^2+^ ions coordinated by six conserved cysteine residues in the Gal4-DNA complex. Mutations on these residues would, therefore, disrupt zinc binding, affecting Gal4 function. (**B**,**C**) depicts noncovalent interaction network of mutated residues in the Gal4-DNA complex. Mutated residues are depicted in green. Hydrogen bonds are depicted as red dotted lines, hydrophobic interactions in green and ring-ring interactions in grey. (Panel **B**) shows residue Tyr40 performing a side-chain to main-chain hydrogen bond and ring interactions. The mutation Y40A is predicted to be highly destabilizing, given the removal of a large portion of the side chain and consequent loss of interactions. (Panel **C**) shows residue Val57, whose mutations are predicted to also affect protein-protein affinity. Val57 establishes a network of hydrophobic and ring interactions that would be lost by the introduction of larger or hydrophilic residues, destabilizing the homodimer. (Panel **D**) shows residue R15, whose mutations are predicted to also affect RNA binding affinity. Arg15 directly interacts with the DNA molecule through a weak polar interaction and hydrogen bond. Mutations to aspartic and glutamic acids, through the introduction of an opposite charge, are predicted to destabilize the region and reducing DNA affinity.

Gal4 DNA recognition and transcriptional activation were known to be mediated by a Zn_2_Cys_6_ binuclear cluster^29^,^30^,^31^,^32^,^33^, and this was confirmed by the experimental structures of Gal4 and its molecular interactions with DNA^28^,^34^,^35^. The pair of Zn^2+^ ions help maintain the fold of the DNA-binding residues and are coordinated by six conserved cysteine residues within the protomer (Fig. 5A). Approximately 90% of mutations (98/110) identified as affecting metal coordination were experimentally observed to lead to reduced growth, with average log-enrichment scores lower than −3. This was consistent with mCSM-metal predictions that the majority of mutations at any of these positions would lead to loss of zinc binding and disrupted function of Gal4, and were strongly correlated with reduced growth (p < 0.001 by two-tailed Z-test).

### Predicted Highly Destabilising Mutations

Using the available crystal structure of the Gal4-DNA complex^28^, the stability change upon mutation was predicted using mCSM-Stability, SDM and DUET. Mutations predicted by mCSM-Stability to be highly destabilizing (ΔΔG < −2 Kcal/mol) were highly associated with reduced growth (p = 0.003 by two-tailed Z-test), with almost 70% of mutations (46/67) presenting average log-enrichment scores lower than −3 (for an example see Fig. 5B). This proportion increased to 85% for mutations (28/33) predicted to have an even greater effect (ΔΔG < −2.5 Kcal/mol).

### Mutations Predicted to Greatly Reduce PPI Affinity

Gal4 function is dependent upon binding DNA as a homodimer^29^,^30^,^34^,^35^. The crystal structure of Gal4 in complex with DNA revealed the dimerisation interface consisted of an intertwined helical bundle^28^. Mutations that would disrupt homodimer formation were therefore predicted would be associated with reduced growth (Fig. 5C). 61% of mutations (19/31) predicted by mCSM-PPI to greatly reduce protein-protein affinity (ΔΔG < −2.0 Kcal/mol) presented a log-enrichment score lower than −3. This proportion increased to 77% (7/9) for the most debilitating mutations (ΔΔG < −2.5 Kcal/mol) that were associated with reduced growth.

### Mutations Predicted to Greatly Reduce DNA Binding Affinity

Gal4 is a transcriptional activator that recognises a consensus 17-base-pair sequence^29^,^30^,^31^,^32^,^33^, with similar sequences located upstream of Gal4-regulated genes. Decreasing the binding affinity to this sequence, for instance by interfering with the interactions established by the wild type (Fig. 5D), would reduce expression of the selectable resistance marker and lead to reduced growth. Mutations predicted by mCSM-NA to greatly disrupt DNA binding (ΔΔG < −2.0 Kcal/mol) were strongly associated with reduced growth (p = 0.004 by two-tailed Z-test), with 74% of mutations (26/35) presenting a log-enrichment score lower than −3.

### Regression and Classification Analysis of All Gal4 Experimental Mutations

None of the individual predictions from each method correlated well with the experimental data (mCSM-Stability ρ = 0.21; SDM ρ = 0.20; DUET ρ = 0.26; mCSM-PPI ρ = 0.21; mCSM-NA ρ = 0.11). This reflects the observation that the mutations exert a range of effects on Gal4, from destabilising the protein, to disrupting its interactions with DNA or within the homodimer.

Using a Random Forest to train a binary classifier using predictions from mCSM-Stability, SDM, DUET, mCSM-PPI and mCSM-NA we were able to correctly classify 81% of the mutations (AUC = 0.86).

**Figure 6:**
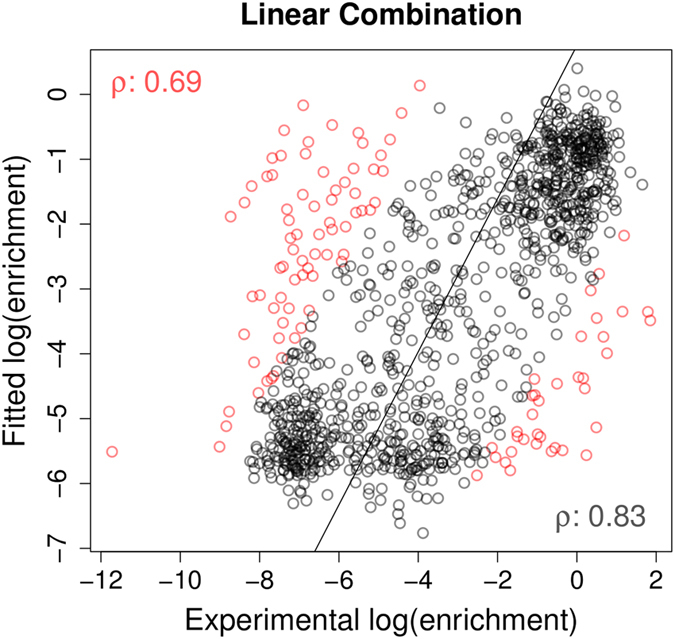
Performance analysis on regression on Gal4 mutations. The graph shows regression results on 10-fold cross validation for the predictive model trained on the complete set of mutations (1083) on Gal4. Fitted log(enrichment) scores using DUET, SDM, mCSM-Stability, mCSM-PPI and mCSM-NA are combined using linear equations compared to the average phenotypic results obtained by Kitzman e*t al.* (2015). Pearson’s correlation coefficient (ρ) is shown in the bottom-right part of each graph and at the top-left after 10% outlier removal. Outliers are depicted in red.

**Figure 7:**
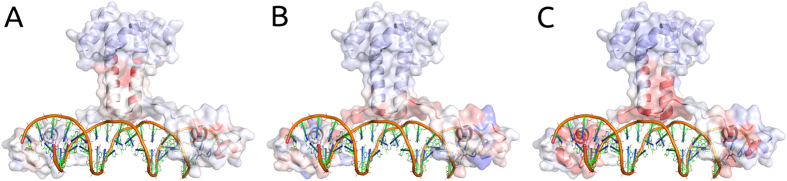
Heatmap of the average predicted and experimental changes upon mutation on Gal4. The figure shows the average prediction per residue in stability (left), DNA affinity (middle) and experimental measurement of enrichment score (right). Residues were coloured in a scale from blue to red indicating the average effect from stabilizing to destabilizing as predicted by mCSM-Stability, mCSM-NA or the degree of reduced cell growth as described experimentally. It is interesting to notice the predicted variability of effects on protein stability and DNA affinity on the structures and how they are together complementary to the experimental phenotype.

A correlation of ρ = 0.69 was obtained on 10-fold cross validation, by linearly combining these predictions using a Regression Model Tree. A regression plot of the obtained model is shown in Fig. 6. A heatmap analysis of the effects of the mutations on protein stability and DNA affinity (Fig. 7) shows the predicted variability of effects on protein stability and DNA affinity on the structures and how they are together complementary to the experimental phenotype. As observed with DBR1, the outlier mutations tended to have very low log-enrichment scores (Figure S6 of Supplementary material).

## Mutations on von Hippel-Lindau disease and risk of renal carcinoma

von Hippel-Lindau (VHL) disease is an inherited condition caused by mutations on the VHL gene which are associated with propensity for tumours, including clear cell renal cell carcinoma (ccRCC). In a recent work, Gossage and colleagues^36^ assembled a database of 121 missense mutations on VHL linked with experimental and clinical data, including associations with ccRCC. This was used to develop a pipeline, Symphony, to study mutations in this protein and predict risk of ccRCC by integrating different methods. From the original data set, 62 mutations were categorized as high risk and the remaining 59 as low risk of ccRCC and were used as training set. An additional set of 173 mutations was used as test set. We have applied our new pipeline to these mutations in order to assess its performance in correlating mutation effects with clinical outcomes (Figure S7).

### Mutations predicted to greatly affect protein stability and protein-protein affinity

**Figure 8:**
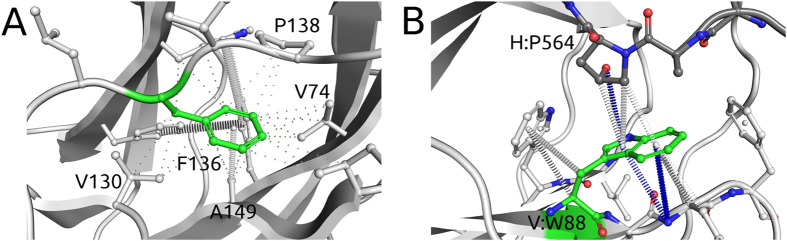
Noncovalent interaction networks in VHL. Mutated residues are depicted in green. Proximal hydrophobic interactions are depicted in small dots, ring-ring interactions in grey and donor-pi interactions in blue. (Panel **A**) shows residue Phe136 performing a dense network of hydrophobic and ring interactions. Mutation to serine is predicted to be highly destabilizing, given the removal of a large portion of the side chain and consequent loss of interactions. Panel B shows residue Trp88, whose mutations are predicted to also affect protein-protein affinity. Trp88 establishes a network of ring interactions, as well as donor-pi interactions within its chain and with the HIF-1α peptide. Mutations to arginine or serine would disrupt these strong interactions, destabilize the region as well as the protein-protein interface, reducing affinity.

The available structure of VHL in complex with Elongin B, Elongin C and a HIF-1α peptide (PDB ID: 1LM8) was used in this study. 77% of mutations (17/22) predicted as highly destabilizing by DUET (ΔΔG < −2.0 Kcal/mol) were associated with high risk of ccRCC, a proportion consistent with mCSM-Stability (76%–16/21) and SDM (76%–32/42). All mutations (3/3) predicted to disrupt the protein-protein complex were classified as high-risk (examples depicted in Fig. 8A,B). This is consistent with the idea that risk of ccRCC is directly related to the impact of the mutation on VHL structure and function.

### Predicting risk of ccRCC for all mutations on VHL

A Random Forest binary classifier was trained using stability and protein-protein affinity change predictions from mCSM-Stability, SDM, DUET and mCSM-PPI. ccRCC risk was predicted with 98% sensitivity and 93% specificity, which is consistent with the results described by Gossage *et al.*^36^. Only one mutation associated with ccRCC was predicted low risk, while most mutation associated with other tumours (pheochromocytoma) and polycythemia were predicted as low-risk for ccRCC, also consistent with what was obtained by Symphony.

## Discussion and Conclusion

Elucidating the molecular mechanisms linking a mutation’s impact with phenotype is very often non-trivial, and functional interpretation of mutation data has consequently lagged behind generation of the data from modern high-throughput techniques. We have presented here a workflow to analyze systematically the structural and functional effects of mutations computationally for three different systems where multiple mutations and phenotypic outcomes have been described. We show that our workflow can predict the relative changes mediated by alterations in protein stability and interactions, so providing the opportunity to understand in greater detail the effects of mutations and how they relate to the phenotypes we observe. Together they illustrate how understanding the effects of mutations on protein stability and interactions with other proteins, nucleic acids and metal ions is important for unravelling the link between mutations and phenotypes. The addition of mCSM-Lig will also enable the effect of mutations on protein-small molecule interactions to be taken into consideration.

Our analysis of the recent saturation mutagenesis results of DBR1 and Gal4 demonstrated a correct classification of over 80% of the mutations. No single computational tool correlated strongly with the phenotypic results of all the mutations from either saturation mutagenesis experiment, which is probably not surprising considering the multitude of effects a mutation may have on a protein’s function. By taking into account these multiple effects, however, a unified model could be generated that performed better than the individual predictions (Table S1). The Spearman correlations were also consistent, and are available in Table S2. To understand the link between mutations in DBR1 and changes in cell growth we needed to consider how the mutations affected protein stability, metal coordination and RNA binding affinity, while the effect of mutations on dimerization of Gal4 also needed to be taken into account in order to explain the phenotypic results. This highlights the importance of understanding the system being studied prior to analysis in order to be able to assess the full range of effects a mutation may have.

The outliers from these predictors were mainly associated with extreme values for reduced cell growth. It is therefore worth noting that the remaining mutations may need to be explained by additional factors, for example through changes in transcription and translation. It is important to consider that a mixture of effects could give rise to observed phenotypic outcomes. This has been important in our analyses of disease causing mutations^17^,^37^,^38^,^39^,^40^, where understanding these effects has been extremely useful for guiding treatment strategies.

For example, our recent analysis of the mutations in the protein homogentisate 1,2-dioxygenase that cause the development of the Mendelian disease alkaptonuria revealed that mutations resulted in either instability of the protomer, disruption of the homo-hexameric structure, or direct modification of catalytic site residues^39^,^40^. This understanding is allowing us to explore whether they may alter a patient’s response to treatments, and opens up avenues of designing specific treatments. Combining predictors into a single model can also be a valuable clinical tool, allowing the rapid analysis of novel mutations. For example, by combining multiple predictions, a classifier (Symphony) was able to identify mutations in VHL associated with renal cell carcinoma with high levels of sensitivity and specificity^36^. Application of the current workflow achieved similar accuracy with fewer predictors, allowing rapid classification of the relative effects of a mutation on VHL’s structure and function, and its relationship to the risk of developing ccRCC. This showed that changes in protein stability and protein-protein interactions were important in order to predict the clinical phenotypes.

Dissecting the effects of mutations is a complicated task, but we present here a computational pipeline capable of explaining experimental data and which provides a promising avenue for understanding the role of mutations in evolution, the emergence and progression of diseases and as a corner stone for guiding current and the next generation of treatments.

## Methods

### Homology modelling of DBR1

Models of apo DBR1 and DBR1 in complex with RNA, comprising residues 2-349, were generated using Modeller^41^ and MacroModel (Schrodinger, New York, NY) using the X-ray crystal structures of apo DCR1 (PDB code: 4PEF^24^) and in complex with a substrate analogue, synthetic RNA that mimics the RNA lariat branchpoint (PDB codes: 4PEH and 4PEI^24^) from *Entamoeba histolytica* (35% sequence identity with human DBR1). The models were then minimized using the MMF94s forcefield in Sybyl-X 2.1.1 (Certara L.P., St Louis, MO), with the final structure having more than 95% of residues in the allowed region of a Ramachandran plot. Following previous approaches^42^,^43^,^44^, a manganese ion was manually added to the active site after comparison with the manganese-bound DCR1 structures indicated the conformation of residues in the manganese-binding motif were identical in the two proteins. The root mean square deviation (RMSD) between the models and the templates was 0.21–0.22 Å. The quality of the models was confirmed with Verify3D^45^ (data not shown). Model structures were examined using Pymol.

### Predicting the structural effects of mutations

The effects of the mutations on the stability of DBR1 and Gal4 were analyzed by mCSM-Stability^11^, SDM^4^ and DUET^12^ using both the apo and ligand-bound models. The predicted changes in stability of DBR1 were also predicted by I-Mutant2^6^, PoPMuSiC 2^8^, Foldx^15^ and AutoMute2^46^, however as they did not show an improved correlation over mCSM or DUET (Table S3) they were not used further in the analysis and we have not presented them here. As part of the SDM predictive method, all the mutants were modeled using Andante^47^; however, these were not needed for the mCSM and DUET predictions. The effects of the mutations on manganese coordination by DBR1 and zinc binding by Gal4 were also assessed by mCSM-metal, which is still under development for other metal classes. The effects of the mutations on the affinity of DBR1 for RNA and Gal4 for DNA were analyzed by mCSM-NA^11^ using the model of the complexes. The effects of the mutations on the affinity of the Gal4 homodimer and the VHL-Elongin B/C-HIF-1α complex were predicted by mCSM-PPI^11^. All the predictions for DBR1 are shown in Table S4.

### Machine learning methods

For classification tasks we used the Random Forest algorithm^26^,^48^ to train predictive models. This is an ensemble-learning method where multiple decisions trees are induced over a random subset of features and decide the output via majority voting. It is considered one of the best and more robust classification algorithms capable of dealing with large data sets. For regression tasks we used Regression Model Trees, specifically the M5P algorithm^27^. The model creates a decision tree that divides the data in subgroups based on the input attributes. A linear regression model is then created within each subgroup. The algorithms used are implemented and available through the Weka toolkit^49^. For both classification and regression experiments, models were evaluated under 10-fold cross validation and also leave-one-out cross validation for DBR1.

### Evaluation metrics for predictive models

Classification models were evaluated based on the Area under ROC curve (AUC). Values for AUC range from 0 to 1. A perfect binary classifier would give an AUC of 1, while a random classifier would render an AUC of 0.5. The correctly classified instances (accuracy = tp+tn/(tp+tn+fp+fn)) as well as sensitivity (tp/(tp+fn)) and specificity (tn/(tn+fp)) were also used when applicable. Regression models were evaluated based on the Pearson’s correlation coefficient. Correlation values range from −1 to 1. A value of 1 denotes perfect positive correlation, −1 a perfect negative correlation, while random variables are expected to give a correlation close to 0. As a standard procedure in machine learning, Pearson correlation coefficients are evaluated on the complete data set and after 10% outlier removal in order to assess the fit of the model to the majority of data points, minimising possible large effects from a small proportion of the data (i.e., giving an estimate of performance in 90% of the data).

## Additional Information

**How to cite this article**: Pires, D. E. V. *et al.* *In silico* functional dissection of saturation mutagenesis: Interpreting the relationship between phenotypes and changes in protein stability, interactions and activity. *Sci. Rep.* **6**, 19848; doi: 10.1038/srep19848 (2016).

## Supplementary Material

Supplementary Information

## Figures and Tables

**Figure 1:**
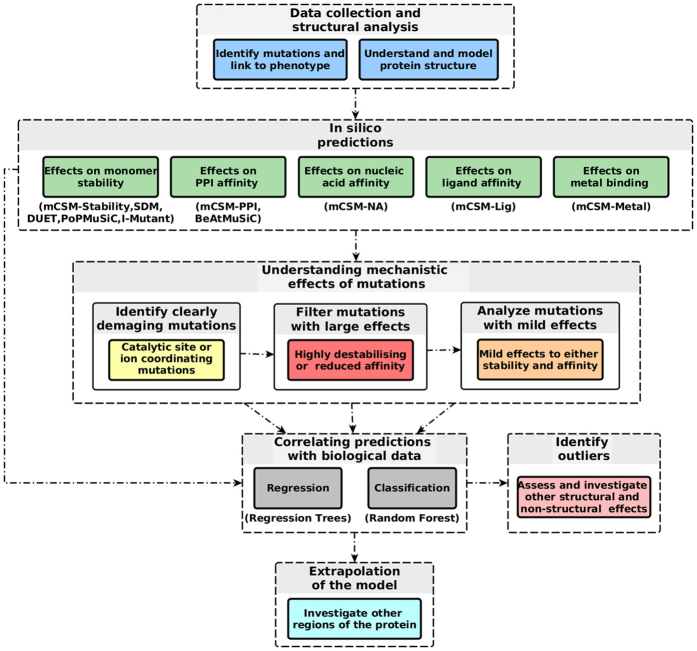
A proposed computational mutation analysis workflow. The figure depicts the proposed methodology workflow which can be divided in the four main steps: data collection and structural analysis, *in silico* (quantitative) prediction of effects of mutations, filtering mutations by their predicted effect, building regression and classification models to link prediction with observed phenotype.
