# Supplementary material for: In silico functional dissection of saturation mutagenesis: Interpreting the relationship between phenotypes and changes in protein stability, interactions and activity
Source: Sci Rep. 2016 Jan 22;6:19848. doi: 10.1038/srep19848 (PMC4726175; doi:10.1038/srep19848)
Supplement: Supplementary Information [file srep19848-s1.pdf]

Supplementary Material

In silico functional dissection of saturation mutagenesis: Interpreting the relationship between phenotypes and changes in protein stability, interactions and activity

Douglas E V Pires[1,2,*], Jing Chen[1], Tom L Blundell[1], David B Ascher[1,*]

[1]Department of Biochemistry, Sanger Building, University of Cambridge, 80 Tennis Court Road, Cambridge, CB2 1GA, UK.

[2]Centro de Pesquisas René Rachou, Fundação Oswaldo Cruz, Avenida Augusto de Lima 1715, Belo Horizonte, 30190-002, Brazil

[*] These authors contributed equally to this work

Correspondence should be addressed to D.B.A: Tel: +44 1223766033; Fax: +44 1223766002; mail: dascher@svi.edu.au; Correspondence may also be addressed to douglas.pires@cpqrr.fiocruz.br

Supplementary Figures

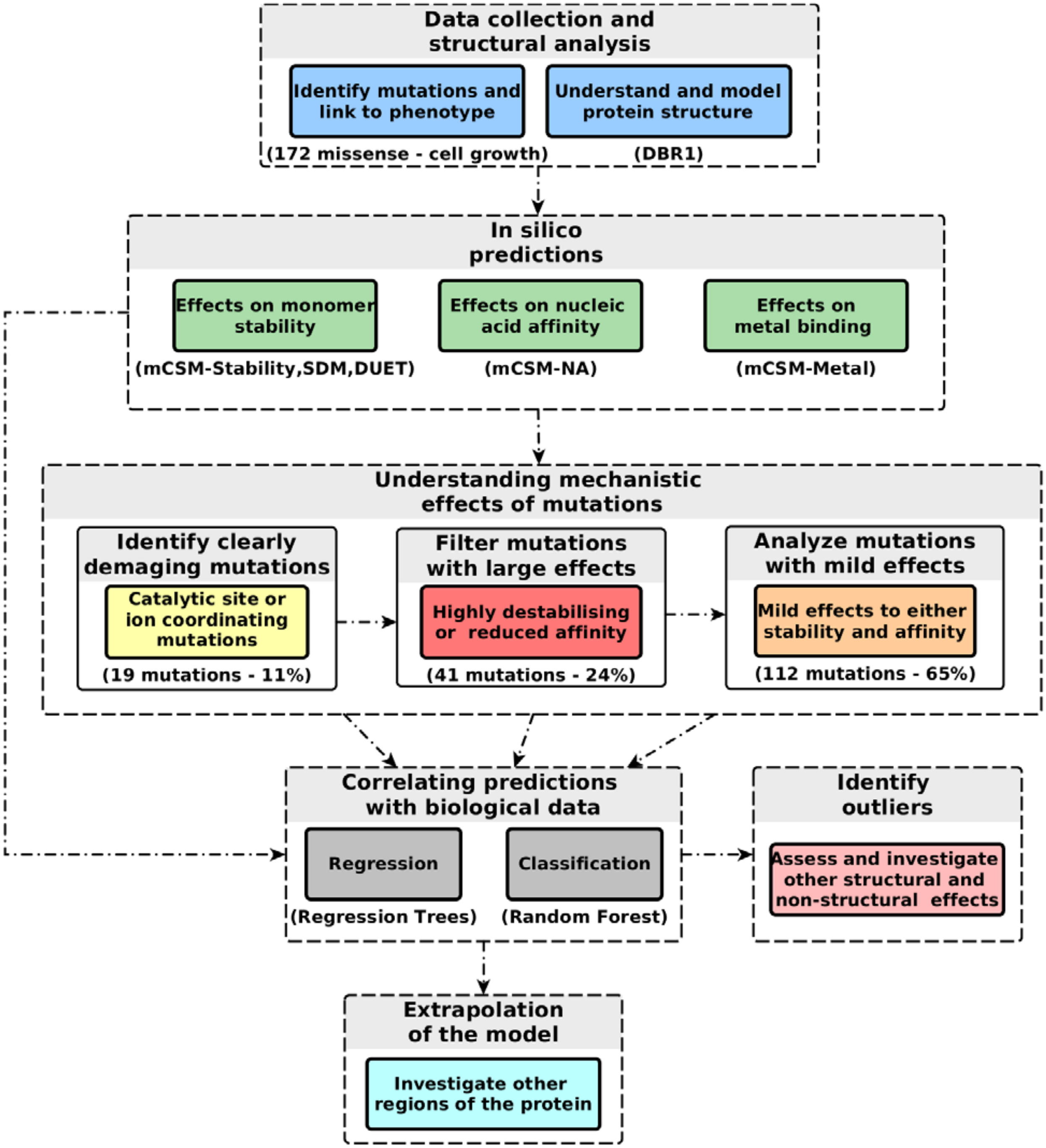

Figure S1 Computational mutation analysis workflow applied to DBR1. The figure depicts the major steps of the methodology workflow applicable to DBR1, which can be divided into: data collection and structural analysis, *in silico* (quantitative) prediction of effects of mutations to stability, RNA binding and metal binding, filtering mutations by their predicted effect, building regression and classification models to link prediction with observed phenotype.

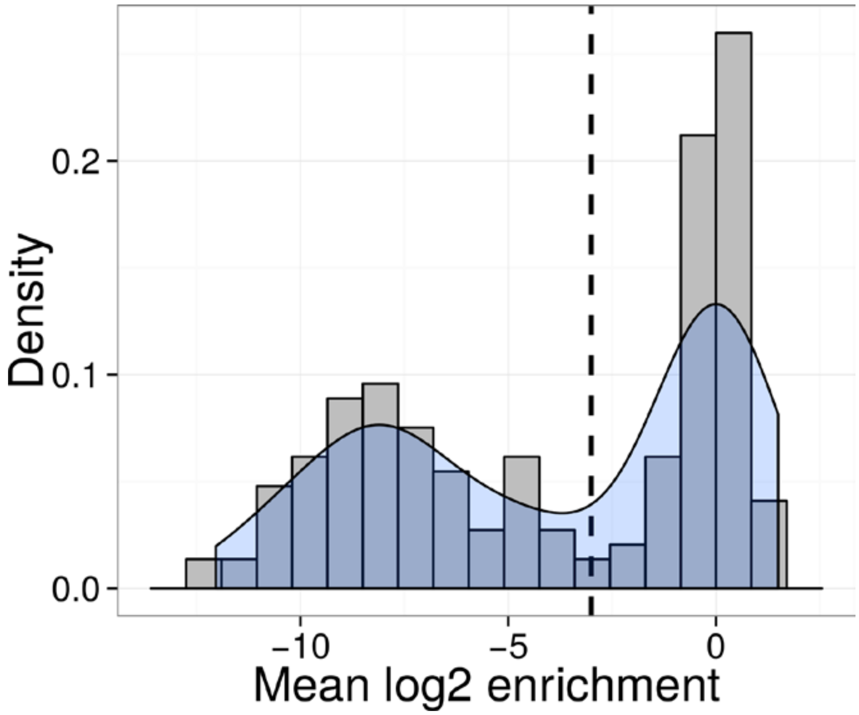

Figure S2 Growth response distribution for the 172 analysed mutations on DBR1 in terms of the average log_2 enrichment score between replicates. The dashed vertical line shows the threshold used to group the mutations into two classes, low or no growth response (on the right) and high response (left group).

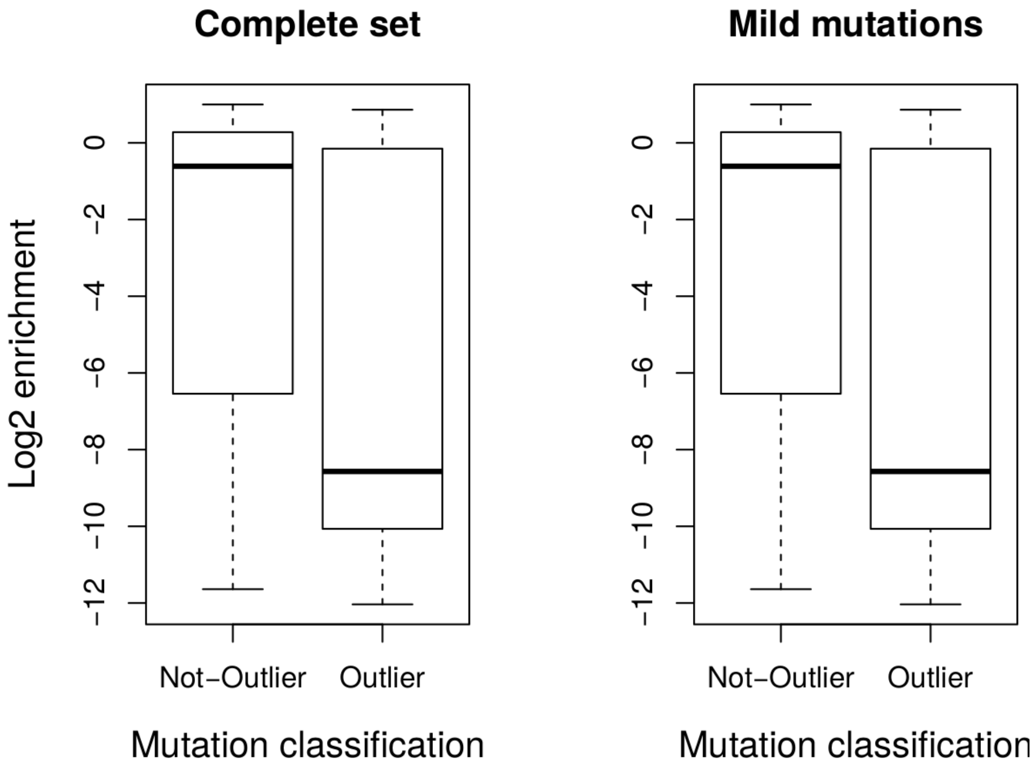

Figure S3. Boxplot of the $\log_2$ enrichment scores between outlier mutations identified on regression tasks and the remaining ones for the complete set and for mild mutations on DBR1. On average, the outliers seem to be mutations with much lower values for growth reduction than the rest of the mutations.

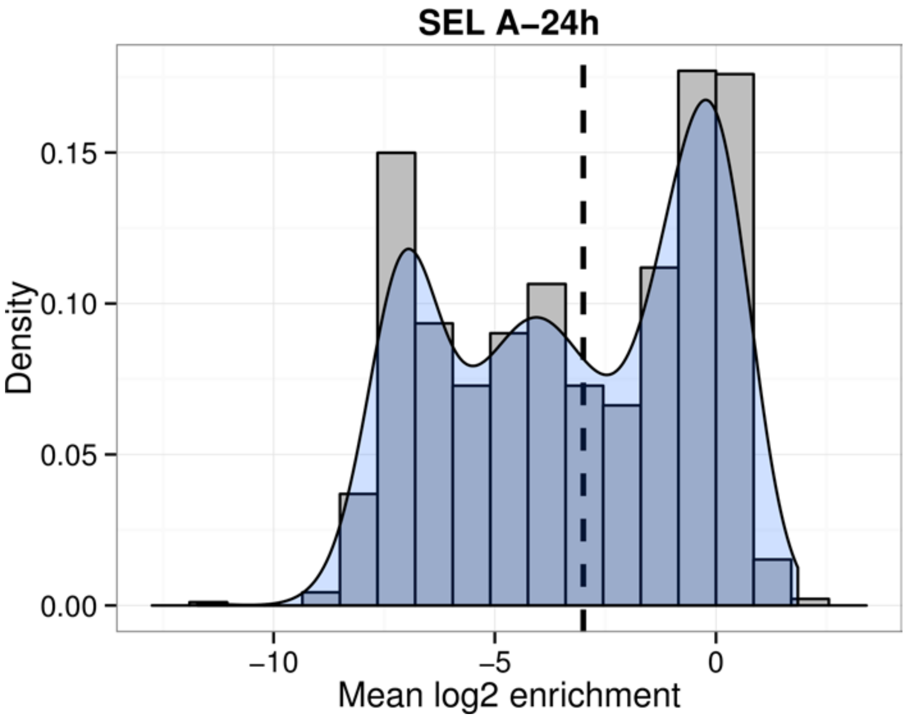

Figure S4 Growth response distribution (average log_2 enrichment score) for the 1083 analysed mutations on Gal4. The dashed vertical line shows the threshold used to group the mutations into two classes, low or no growth response (on the right) and high response (left group).

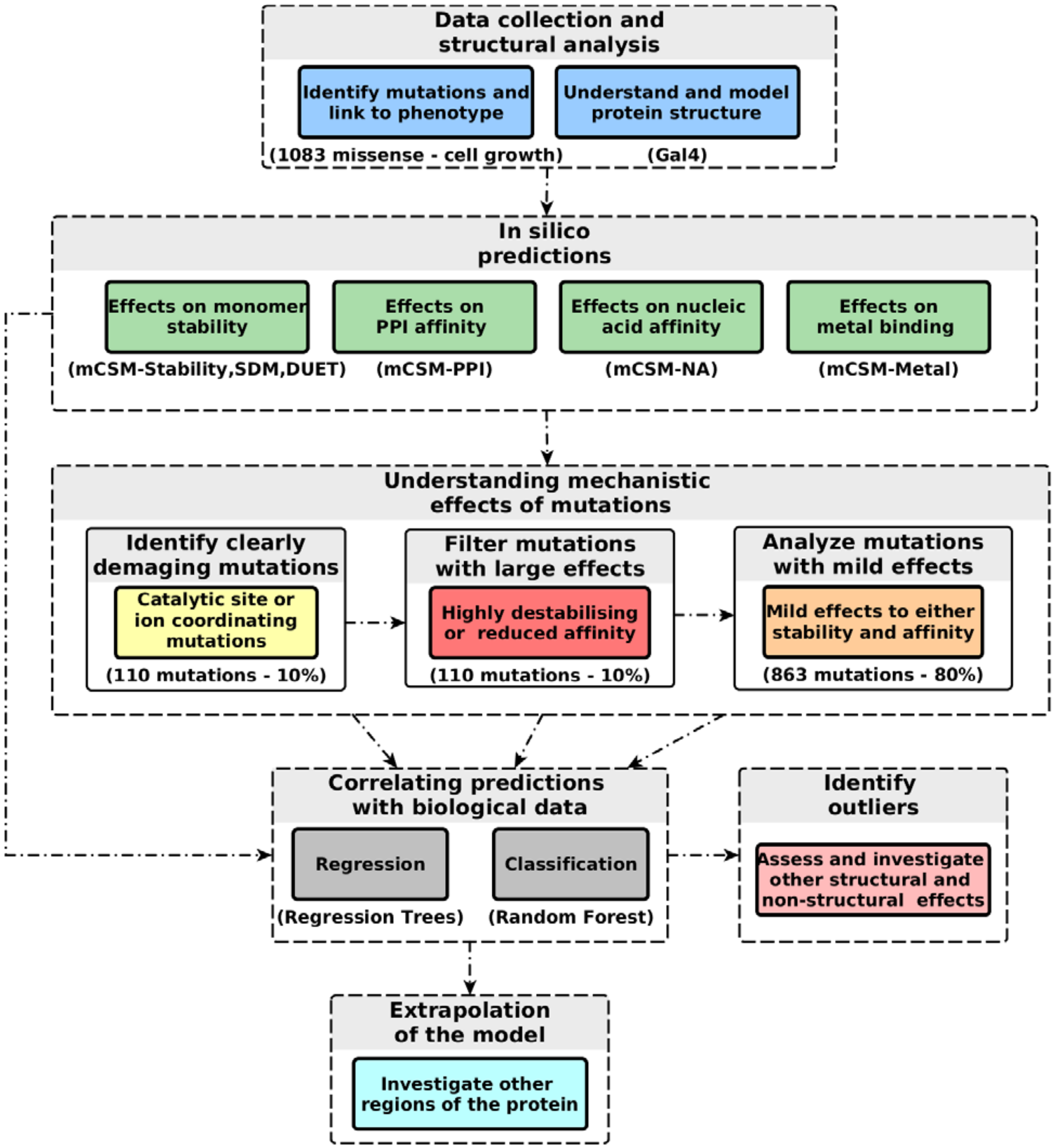

Figure S5 Computational mutation analysis workflow applied to Gal4. The figure depicts the major steps of the methodology workflow applicable to Gal4, which can be divided into: data collection and structural analysis, *in silico* (quantitative) prediction of effects of mutations to stability, DNA binding, protein-protien affinity and metal binding, filtering mutations by their predicted effect, building regression and classification models to link prediction with observed phenotype.

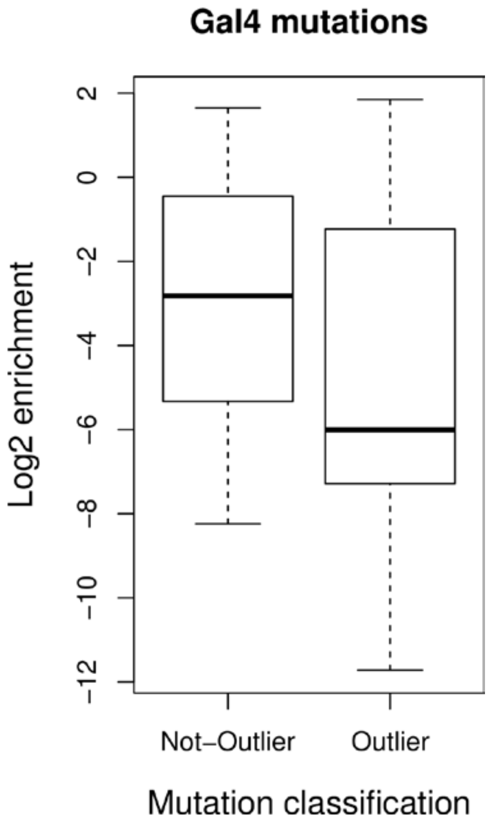

Figure S6. Boxplot of the $\log_2$ enrichment scores between outlier mutations identified on regression tasks and the remaining ones for the complete set and for mild mutations on Gal4. On average, the outliers seem to be mutations with much lower values for growth reduction than the rest of the mutations.

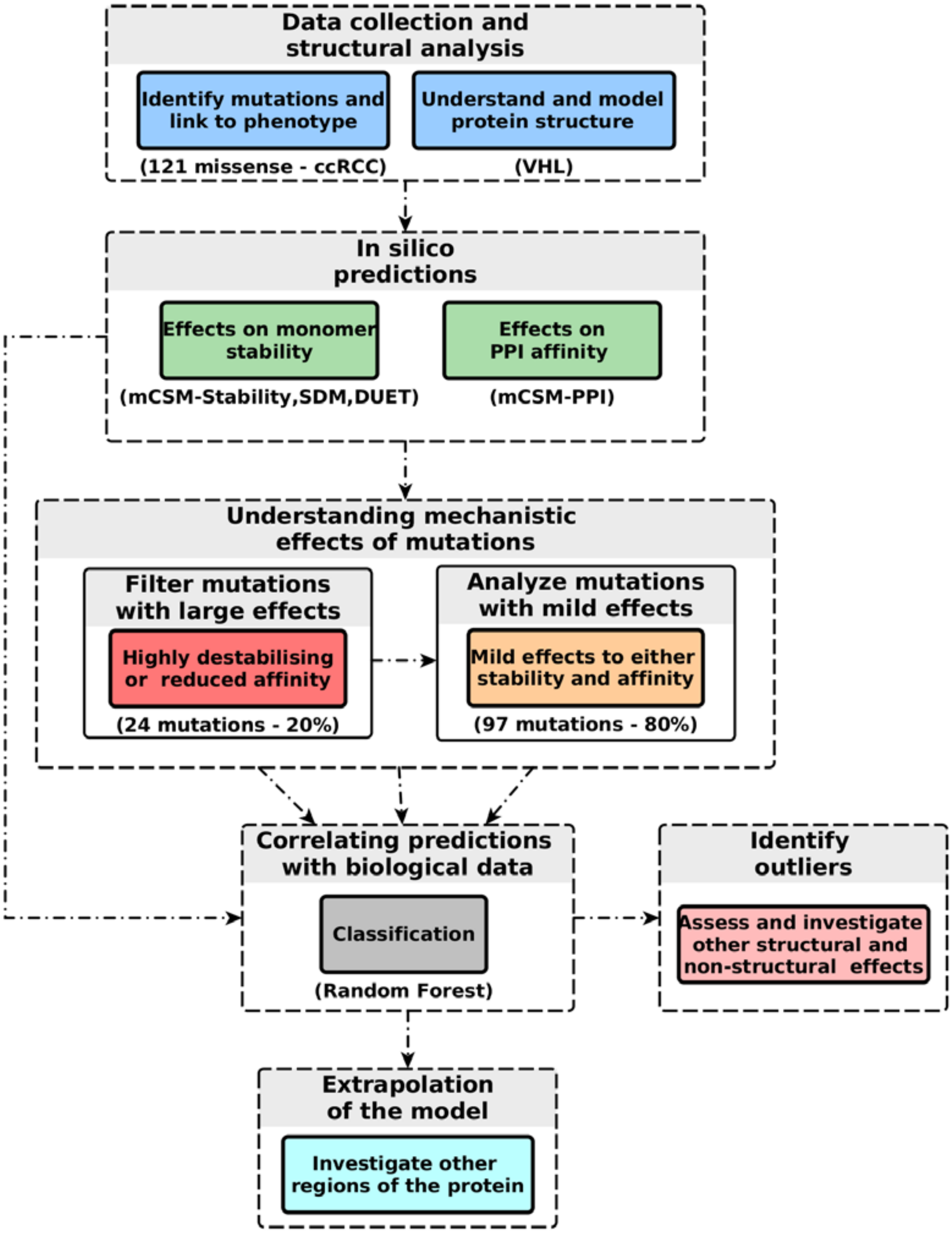

Figure S7 Computational mutation analysis workflow applied to VHL. The figure depicts the major steps of the methodology workflow applicable to VHL, which can be divided into: data collection and structural analysis, *in silico* (quantitative) prediction of effects of mutations to stability, and protein-protien affinity, filtering mutations by their predicted effect, building classification models to link prediction with observed clinical outcome.

Supplementary Tables

Table S1. Pearson's correlation coefficients of individual stability and affinity change predictions and for the linear regression models for DBR1 and Gal4.

	DBR1 mild mutations	DBR1 mild mutations (-10% outliers)	All DBR1 mutations	All DBR1 mutations (-10% outliers)	All Gal4 mutations	All Gal4 mutations (-10% outliers)
mCSM-Stability	0.22	0.22	<0.1	<0.1	0.21	0.36
mCSM-NA	0.48	0.54	0.35	0.54	0.11	0.22
mCSM-PPI	N/A	N/A	N/A	N/A	0.21	0.35
Regression model	0.56	0.71*	0.65*	0.81*	0.69*	0.83*

*Denotes a significantly higher performance for the regression model compared to each of the individual methods by a Steigers's Z-test (two-tailed $p < 0.05$).

Table S2. Spearman rank correlation coefficients of final regression models for DBR1 and Gal4.

	Complete data set	After 10% outliers removal
DBR1 (mild mutations)	0.45	0.56
DBR1 (all mutations)	0.59	0.72
Gal4 a(all mutations)	0.67	0.80

Table S3. Correlation between predicted stability changes upon mutation by other methods and log enrichment scores on DBR1.

**Method**	**Pearson Correlation Coefficient**
PoPMuSiC 2	-0.040
I-Mutant 2	-0.155
FoldX	-0.155
AUTOMUTE	-0.003

Table S4. DBR1 mutations and predicted changes in stability and RNA binding affinity.

**MUTATION**	**AVG. log(enrichment)**<br>From Findlay et al., (2014)	**mCSM_Stability**<br>(ΔΔG kcal/mol)	**DUET**<br>(ΔΔG kcal/mol)	**SDM**<br>(ΔΔG kcal/mol)	**mCSM_NA**<br>(ΔΔG kcal/mol)
N84A	-10.7543	-0.604	-0.646	1.48	0.356
N84C	-8.076	-0.011	-0.057	1.63	0.972
N84D	-9.78325	-1.497	-1.503	-0.11	0.039
N84E	-10.0645	-1.291	-1.285	-0.42	0.042
N84F	-10.0192	-0.07	-0.207	2.2	0.491
N84G	-4.41012	-0.99	-1.257	-1.31	0.354
N84H	-4.4005	-0.924	-1.092	0.81	0.06
N84I	-8.77258	0.422	0.408	1.76	0.365
N84K	-4.8035	-0.42	-0.316	-0.9	0.776
N84L	-8.66092	0.422	0.4	1.19	0.365
N84M	-9.35	0.728	0.542	1.48	0.362
N84P	-10.8435	0.108	0.086	1.14	0.362
N84Q	-8.62725	-0.764	-0.714	-0.27	0.508
N84R	-8.6664	-0.874	-0.757	0.63	0.574
N84S	-8.0185	-1.165	-1.153	-0.88	0.969
N84T	-8.56888	-0.799	-0.753	-0.51	0.972
N84V	-12.0346	0.108	0.09	1.42	0.362
N84W	-6.03	-0.223	-0.306	4.23	0.559
N84Y	-6.132	0.137	0.037	2.55	0.491
H85A	-6.54175	-0.971	-1.038	1.4	1.122
H85C	-8.278	-0.341	-0.334	2.14	1.738
H85D	-8.72125	-1.223	-1.245	0.16	0.805
H85E	-6.58675	-1.1	-1.199	-1.43	0.807
H85F	-11.6402	0.551	0.4	0.18	1.257
H85G	-8.6195	-1.309	-1.589	-2.54	1.119
H85I	-10.5615	0	-0.166	-0.45	1.13

H85K	-10.443	-0.68	-0.786	-2.9	1.542
H85L	-10.5171	0	-0.096	1.3	1.13
H85M	-9.3065	-0.127	-0.38	0.08	1.128
H85N	-6.38025	-0.97	-1.129	-1.45	1.271
H85P	-8.00812	-0.311	-0.547	-1.55	1.128
H85Q	-4.60575	-0.617	-0.759	-1.66	1.274
H85R	-8.5957	-1.15	-1.187	-1.24	1.34
H85S	-8.13917	-0.951	-1.114	-2.12	1.735
H85T	-5.49483	-0.742	-0.859	-1.17	1.738
H85V	-7.50017	-0.311	-0.462	0.12	1.128
H85W	-11.9565	0.686	0.561	2.04	1.325
H85Y	-8.25	0.66	0.525	0.2	1.257
E86A	-4.82825	-2.508	-2.576	0.78	2.758
E86C	-8.062	-1.601	-1.635	0.84	3.374
E86D	0.27975	-1.734	-1.934	-1.06	2.441
E86F	-10.6537	-2.58	-2.446	2.46	2.893
E86G	-7.80487	-2.816	-3.291	-1.97	2.756
E86H	-8.284	-2.222	-2.059	2.39	2.462
E86I	-9.59567	-1.613	-1.531	3.78	2.767
E86K	-7.7655	-2.084	-2.291	-1.74	3.178
E86L	-8.1825	-1.613	-1.567	2.47	2.767
E86M	-11.718	-0.834	-0.832	4.32	2.764
E86N	-0.43925	-2.109	-2.142	0.24	2.907
E86P	-9.4505	-1.902	-2.309	-1.79	2.764
E86Q	-7.368	-1.906	-1.952	0.09	2.91
E86R	-7.5337	-1.817	-1.759	-0.15	2.976
E86S	-8.39492	-2.869	-2.965	-0.17	3.371
E86T	-9.61625	-2.55	-2.635	0.23	3.374
E86V	-7.19037	-1.902	-1.856	3.33	2.764
E86W	-9.917	-2.611	-2.183	0.7	2.961
E86Y	-6.999	-2.412	-2.285	1.7	2.893
A87D	-7.1875	-2.915	-3.192	-2.28	2.212

A87G	-1.287	-1.476	-1.975	-4.78	2.527
A87P	0.1945	-0.428	-0.762	-3.62	2.535
A87S	1.2885	-1.81	-2.029	-2.71	3.143
A87T	-4.5235	-1.586	-1.819	-3.28	3.145
A87V	-0.2275	-0.428	-0.438	-0.53	2.535
S88A	-3.9435	-0.754	-0.088	2.85	0.138
S88L	-0.3725	-0.016	0.594	2.43	0.147
S88P	0.5255	-0.227	-0.388	-0.96	0.144
S88T	-2.336	-0.858	-0.472	0.54	0.754
N89H	-1.028	-0.943	-0.873	3.18	0.198
N89I	-0.4665	0.382	0.614	2.71	0.093
N89K	-0.054	-0.317	-0.152	-0.48	-0.116
N89S	-0.007	-1.217	-1.162	-0.58	0.042
N89T	0.2815	-0.805	-0.708	-0.12	-0.012
N89Y	-0.0265	0.117	-0.18	1.21	0.051
H90D	-9.9325	-2.178	-2.283	-1.73	0.089
H90L	0.752	-0.844	-0.748	2.15	0.936
H90N	-0.5265	-1.918	-2.187	-4.09	0.46
H90P	-7.018	-1.249	-1.541	-2.6	0.99
H90Q	-0.34825	-1.512	-1.648	-1.51	0.406
H90R	-7.2685	-0.994	-1.044	-1.95	0.73
H90Y	-1.28	0.2	0.142	-0.66	0.894
L91F	-0.368	-1.484	-1.644	-0.34	0.978
L91M	-0.18	-1.031	-1.117	-0.39	1.074
L91S	0.1295	-3.044	-3.199	-3.49	0.969
L91W	-8.5415	-2.031	-1.945	0.42	0.877
Q92E	-0.133	-1.215	-1.156	0.15	-1.109
Q92H	0.7	-0.985	-1.087	1.37	-0.103
Q92L	-0.09	0.608	0.873	3.08	-0.208
Q92P	-8.509	0.218	-0.027	-2.97	-0.155
Q92R	-0.623	-0.87	-0.744	-0.43	-0.414
E93A	0.8315	-1.062	-1.112	0.5	1.719

E93D	-3.48275	-1.566	-1.694	-1.88	0.711
E93G	-0.613	-1.494	-1.873	-3.59	1.772
E93K	-5.5725	-1.589	-1.607	-0.92	1.349
E93Q	0.285	-1.597	-1.575	-0.15	1.028
E93V	0.8645	-0.231	-0.201	0.49	1.612
L94F	-1.32775	-1.479	-1.772	0.21	-1.076
L94I	0.6695	-0.896	-0.948	-0.28	-1.034
L94S	-1.6275	-2.674	-2.882	-2.25	-1.085
L94V	-0.5175	-1.387	-1.637	-1.32	-0.98
P95H	-0.2375	-2.065	-2.191	1.42	0.807
P95L	0.379	-0.522	-0.385	2.48	0.324
P95R	0.121	-1.221	-1.231	1.37	1.11
P95S	0.01	-2.208	-2.315	-0.21	0.646
Y96C	0.571	-0.392	-0.229	2.75	0.191
Y96D	-0.9475	0.721	0.868	0.99	-0.157
Y96F	0.5805	-0.385	-0.209	0.03	0.083
Y96H	0.4485	0.119	0.269	-0.25	0.471
Y96N	0.279	-0.67	-0.644	-0.15	0.371
Y96S	0.705	-1.161	-1.072	0.04	0.31
G97A	0.44	0.545	0.298	-2.19	0.594
G97C	-1.4305	0.898	0.516	-3.08	0.829
G97D	-6.6215	-0.366	-0.665	-4.31	0.482
G97R	-6.543	-0.01	-0.208	-3.71	1.412
G97S	-3.4245	0.071	-0.187	-4.27	0.948
G97V	-5.682	1.249	1.058	-1.02	0.615
G98A	-0.1075	0.457	0.745	1.65	0.707
G98C	-5.9125	0.997	1.269	2.42	0.943
G98D	-2.557	-1.072	-0.854	0.18	0.595
G98R	-10.8865	-0.315	0.039	2.16	1.526
G98S	0.5515	-0.013	0.335	1.04	1.062
G98V	-7.607	1.233	1.6	3.1	0.729
W99C	-0.38425	-0.212	-0.128	2.04	0.399

W99G	-10.1265	-2.439	-2.544	-5.73	0.153
W99L	0.5495	-1.393	-1.488	-2.41	0.196
W99R	-7.80175	-1.731	-1.573	-0.79	0.982
W99S	-4.8345	-2.181	-2.122	-2.55	0.518
V100A	-0.589	-1.52	-1.775	-2.73	-1.135
V100E	-7.199	-2.35	-2.563	-3.8	-2.197
V100G	0.6445	-2.024	-2.475	-5.09	-1.082
V100L	0.021	-0.067	0.11	-0.79	-1.296
V100M	0.6725	-0.173	-0.174	-0.88	-1.242
A101G	-1.8985	-0.992	-1.072	-1.87	0.422
A101P	-8.999	0.309	0.303	-2.44	0.519
A101S	0.176	-1.353	-1.306	-2.09	0.77
A101T	0.4985	-1.007	-0.895	-1.46	0.802
A101V	-4.196	0.309	0.558	1.88	0.519
P102A	0.9835	-1.322	-1.332	-0.77	-0.033
P102L	-0.006	-0.408	-0.387	-1.04	0.065
P102Q	0.596	-1.081	-0.968	0.06	-0.207
P102R	0.531	-0.688	-0.688	-1.03	0.733
P102S	-0.554	-1.525	-1.465	-0.5	0.283
P102T	-0.3865	-1.35	-1.293	-0.9	0.315
N103D	0.1985	-1.296	-1.331	-0.11	-0.232
N103H	-1.7785	-0.743	-0.864	0.94	0.396
N103I	1.0005	0.276	0.404	2.64	-0.087
N103K	-0.6065	0.009	0.103	-0.38	0.29
N103T	0.6965	-0.555	-0.525	-0.31	0.245
N103Y	-0.021	0.128	-0.122	1.44	0.201
I104F	-0.527	-1.374	-1.489	-0.76	0.77
I104L	0.3365	-0.537	-0.399	-0.59	0.675
I104M	-0.1505	-0.581	-0.762	-1	0.727
I104N	-0.4365	-2.281	-2.412	-5.26	1.058
I104S	-4.291	-2.709	-2.919	-4.99	0.996
I104T	-3.34	-2.242	-2.408	-4.34	1.007

I104V	0.652	-1.106	-1.033	-0.83	0.664
Y105D	-4.7025	-1.906	-2.147	-4.34	0.412
Y105F	0.495	-0.583	-0.486	1.01	0.651
Y105H	0.4855	-1.637	-1.739	-2.04	1.039
Y105N	0.9795	-1.737	-1.977	-4.75	0.939
Y105S	-1.0175	-1.747	-2.003	-4.86	0.878
Y106C	-0.9445	-0.155	0.038	0.98	0.648
Y106D	-7.067	-2.585	-2.844	-4.34	0.3
Y106F	0.371	-0.836	-0.438	1.01	0.54
Y106H	-0.389	-2.091	-2.255	-2.29	0.928
Y106N	0.3285	-1.804	-2.049	-4.75	0.828
Y106S	-0.7565	-1.976	-2.222	-4.25	0.767
L107F	0.14575	-1.277	-1.5	-0.31	0.831
L107I	0.384	-0.683	-0.655	-0.36	0.736
L107S	1.496	-2.32	-2.55	-4.01	1.058
L107V	-0.3525	-1.188	-1.419	-1.91	0.725
G108C	-6.635	-1.312	-1.62	-3.08	0.791
G108S	-7.8265	-1.381	-1.566	-4.28	0.91
